# High Dose Gamma Radiation Selectively Reduces GABAA-slow Inhibition

**DOI:** 10.7759/cureus.1076

**Published:** 2017-03-04

**Authors:** Beza A Dagne, Melis K Sunay, Noëlie S Cayla, Yi-Bing Ouyang, Susan J Knox, Rona G Giffard, John R. Adler, Bruce Maciver

**Affiliations:** 1 Anesthesia, Stanford University School of Medicine; 2 Department of Radiation Oncology, Stanford University Medical Center; 3 Department of Neurosurgery, Stanford University School of Medicine

**Keywords:** radiomodulation, synaptic inhibition, gamma, pain therapy, brain slice, synapse, gaba, inhibition

## Abstract

Studies on the effects of gamma radiation on brain tissue have produced markedly differing results, ranging from little effect to major pathology, following irradiation. The present study used control-matched animals to compare effects on a well characterized brain region following gamma irradiation. Male Sprague-Dawley rats were exposed to 60 Gy of whole brain gamma radiation and, after 24-hours, 48-hours, and one-week periods, hippocampal brain slices were isolated and measured for anatomical and physiological differences. There were no major changes observed in tissue appearance or evoked synaptic responses at any post-irradiation time point. However, exposure to 60 Gy of irradiation resulted in a small, but statistically significant (14% change; ANOVA p < 0.005; n = 9) reduction in synaptic inhibition seen at 100 ms, indicating a selective depression of the gamma-aminobutyric acid (GABA_A)_ slow form of inhibition. Population spike (PS) amplitudes also transiently declined by ~ 10% (p < 0.005; n = 9) when comparing the 24-hour group to sham group. Effects on PS amplitude recovered to baseline 48 hour and one week later. There were no obvious negative pathological effects; however, a subtle depression in circuit level inhibition was observed and provides evidence for ‘radiomodulation’ of brain circuits.

## Introduction

Stereotactic radiosurgery (SRS) using gamma radiation is currently used to treat trigeminal neuralgia, primary brain tumors and metastases [1-2] and is being explored as a treatment for severe depression, obsessive-compulsive disorder (OCD), movement disorders as well as for other refractory psychiatric and central nervous system (CNS) pathologies. However, the impact of such a treatment on neurons and synaptic circuitry is poorly understood. There have been a number of studies published on how the brain responds to radiosurgery levels of radiation, yet the results have had a mixture of conclusions and the neuronal effects of irradiation remain unclear.

Some in vivo and in vitro studies suggest high dose SRS can cause neurotoxicity. Previous studies have demonstrated that neuronal degeneration can occur at 70 to 200 Gy and, at the higher doses (150 and 200 Gy), animals showed histological signs of necrosis, edema, and vessel wall thickening [3]. However, the same study, which exposed the animal’s right frontal lobe to stereotactic irradiation, with a single 4 mm isocenter, showed little to no histological changes in animals irradiated with 60 Gy or less. Pathological changes, measured by histological assessments of neuronal, glial, and vascular changes within the target and surrounding brain volume, were not present in animals that received 30, 40, 50, or 60 Gy. However, in vitro studies looking at the volume of brain subregions after irradiation have shown that neuronal death can occur after irradiation [4]. Similarily, a dose-dependent loss of neurons is noticed when looking at both neurogenesis and cellular differentiation of neurons in the hippocampal region of the brain [5]. Also, a study looking at the oxidative damage of brain tissue after a prolonged exposure (21 days before brain tissue isolation) of low-dose (0.04 Gy) radiation exposure observed an increase in oxidative stress biomarkers that could possibly lead to brain injury [6]. In contrast, a more recent histological study has shown that tissue integrity or neuron distribution was not changed a year after irradiation of 45 Gy delivered with 5 Gy fractions twice per week for 4.5 weeks [7].

There is even less clarity about post radiation outcomes in electrophysiological studies. Early studies looking at physiological responses have shown drastic changes to brain physiology at minimal dosage of irradiation. In vitro exposure to radiation with as low as 5-10 Gy appeared to significantly impair neuronal function in the hippocampal area at the synaptic level [8] as did more moderate doses of 25–75 Gy [9-10]. More specifically, the damage noted in these studies did not involve cell death, but instead there was an alteration of neuronal excitability observed as a decrease in the orthodromic population spike caused by both synaptic and postsynaptic damage in a dose and dose rate dependent manner. Both acute and long-term negative impacts on synaptic efficacy (ability of the tissue to transmit synaptic potentials) and spike generation (ability of those synaptic potentials to generate spikes) were observed. Additionally, an electrocorticogram brain activity study on rats showed changes in the pattern of recordings, particularly of the theta waves, at 24 hours and persisting to 90 days after exposure to 18 Gy of gamma radiation [11]. The scarcity and inconsistent findings from earlier electrophysiological studies was taken into consideration for the experimental design in the present study.

Behavioral studies have also shown a range of results with regard to the neurotoxicity of irradiation. Some of these studies looked at locomotive activity, place recognition, and object placement capabilities of irradiated animals. Neurocognitive decline associated with cranial irradiation can involve damage to the neural stem cell niche in the subgranular zone (SGZ) leading to a decline in neurogenesis and changes in the number of microglia resulting in hippocampal inflammation ([12]). Some of the studies found adult neurogenesis was arrested completely after brain irradiation ranging from 3 Gy to 20 Gy; and behavioral as well as cognitive impairments were noted as well [13-16]. Some of these impairments include deficits in sensorimotor function [17], novel object recognition tasks [18], associative learning [19], and reversal learning [20]. Hippocampal-dependent spatial memory deficits may be related to a disruption in neurogenesis [13] that could contribute to longer-term effects. In most studies, the observed deficits recover within weeks or months after irradiation. However, other behavioral assessment studies done on irradiated animals revealed persistent and progressive deficits in hippocampal dependent learning [21]. To further complicate the matter, there have been studies that have shown no cognitive deficits following irradiation in tests including water maze performance (hippocampal dependent learning) [14], reversal learning [22], and object recognition [14, 21]. In contrast, one study has even shown an improvement in behavioral performance in irradiated animals [23].

Other studies have also brought up results showing a complex pattern of neurobehavioral responses that make it very clear that further studies are needed to elucidate what may be going on in the brain after radiation exposure. One such study looking at cognitive deficits after a whole body exposure of gamma-radiation showed that only the low-dose exposure group (2 Gy) and not the high dose group (5 Gy and 8 Gy) had observed dysfunction in their short-term memory [24]. The same study concluded that damage to long-term memory was observed only at the highest dose group of 8 Gy. This group also used magnetic resonance imaging (MRI) and diffusion tensor imaging (DTI) to show that the hippocampus was one of the few areas in the brain heavily damaged by the whole brain irradiation. This and the other studies showing hippocampal-related radiation damage gives us a sense that the hippocampus is an important brain region for studies in this field.

In summary, histological, behavioral, and electrophysiological studies have shown variable results regarding the effect of irradiation on pathological changes in brain tissue. In large part these variable results likely come about from methodological differences between studies. Therefore, the effects of ionizing radiation on brain tissue needs to be further elucidated, and an interdisciplinary approach needs to be used to understand the underlying effects of irradiation on the brain. This paper combines both electrophysiological and histochemical methodology to explore the effects of irradiation at the neuronal and synaptic level, as a test for the hypothesis that gamma irradiation could induce non-toxic, radiomodulatory effects on synaptic transmission.

## Materials and methods

### Rat brain radiation

All experiments described herein were pre-approved by the Stanford University Administrative Panel for Laboratory Animal Care. Male Sprague Dawley rats, 26 days old and 80–100 gram in body weight, were purchased from Charles River Laboratories. The rats were tested and found to be negative for specific pathogens. The rats were normally bred and maintained under specific pathogen-free conditions, and sterilized food and water were available ad libitum. The rats were randomly assigned to two groups: sham irradiation control and 60 Gy gamma irradiation. This dose of radiation was chosen to best mimic doses used in humans for experimental studies of efficacy in treating psychiatric conditions. The rats were anesthetized with an intraperitoneal injection of a cocktail solution of ketamine (70 mg/kg) and xylazine (7 mg/kg) immediately before irradiation. The anesthetized rats were then placed in individual lead boxes with the upper part of the head protruding through a cutout window at the front of each box. Radiation was delivered using a Philips RT-250 200 kVp X-ray unit (Philips, MA, USA) (12.5 mA; half-value layer, 1.0 mm Cu) at a dose rate of 140 cGy/min. The whole brain was locally irradiated with a single dose of 60 Gy. After irradiation, the rats were returned to their cage for recovery.

### Brain isolation and sectioning

Both irradiated (n = 22) and sham animals (n = 31) were studied using the same protocol, including technique and surgeon for the harvesting of and preparation of brain slices. The irradiated animals were euthanized for brain slice isolation 24 hr (n = 9), 48 hr (n = 8), or one week (n = 5) after exposure to gamma radiation. The animals were anesthetized using isoflurane (three percent within a course of five minutes) and euthanized by way of decapitation. The brain was then removed from the skull while being continuously perfused by artificial cerebrospinal fluid (ACSF) consisting of the following concentrations: 124 mM NaCl, 3.5 mM KCl, 1.25 mM NaH_2_PO4, 2 mM MgSO_4_, 2mM CaCl_2_, 26 mM NaHCO_3_ and 10 mM glucose. The pH of the ACSF was 7.4 following saturation with O_2_/CO_2_ (95/5%), to mimic the ideal physiological pH level and oxygen saturation. The brain slices from these rats were prepared in accordance to National Institute of Health guidelines and procedures approved by Stanford University’s Institutional Animal Care Committee.

The brain slices were prepared using a vibratome (VT1000S, Leica Microsystems, Wetzlar, Germany) to cut transverse 400 μm thick hippocampal slices. These axial sections were further cut sagittally and placed on nitrocellulose membrane filter papers. Slices on the filter paper were then placed inside a chamber where a continuous perfusion of ACSF liquid was provided along with a humidified gas phase carbogen (95% O_2_/ 5% CO_2_). The slices were allowed to stabilize in this perfusion chamber for at least an hour before experiments were conducted. This chamber was kept at room temperature (22°C) and the experiments were also conducted at room temperature. For the experimental portion of the study, brain slices were moved to a recording chamber where they were continuously perfused at 2–3 ml/min with ACSF bubbled with carbogen (95% O_2_/ 5% CO_2_).

### Electrophysiolgical recordings

**Figure 1:**
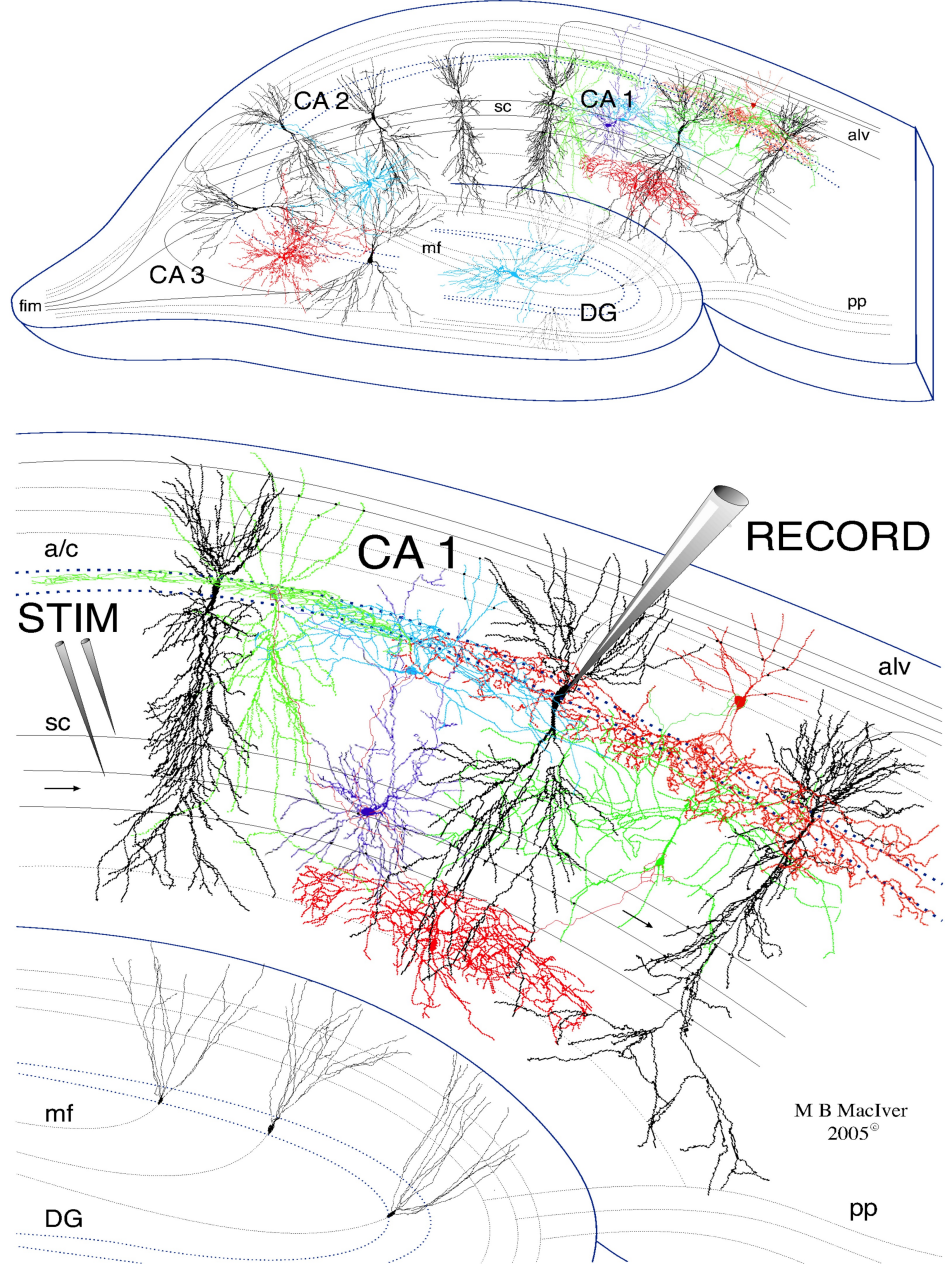
Drawing of a 400-µm thick rat hippocampal slice used for electrophysiological recordings. Three major excitatory pathways of the hippocampal formation are depicted: the perforant pathway (pp) originating from the entorhinal cortex and projecting to granule cells of the dentate gyrus (DG), the mossy fiber pathway (mf) which is comprised of the granule cell axons extending to the pyramidal cells of the CA3, and finally the Schaffer collaterals (sc) make glutamate-mediated excitatory synapses with dendrites of the CA1 pyramidal cells and inhibitory interneurons. On the bottom, an enlarged view showing the relative positions of recording and stimulating electrodes in the area of interest: the CA1 region. Extracellular electrophysiological recordings are produced by stimulating Schaffer-collateral (sc) fibers using a bipolar tungsten microelectrode. Field potentials were recorded by placing a recording micropipette filled with ACSF at the border between stratum oriens and stratum pyramidale, near the axonal output side of the CA1 cell body layer.

**Figure 2:**
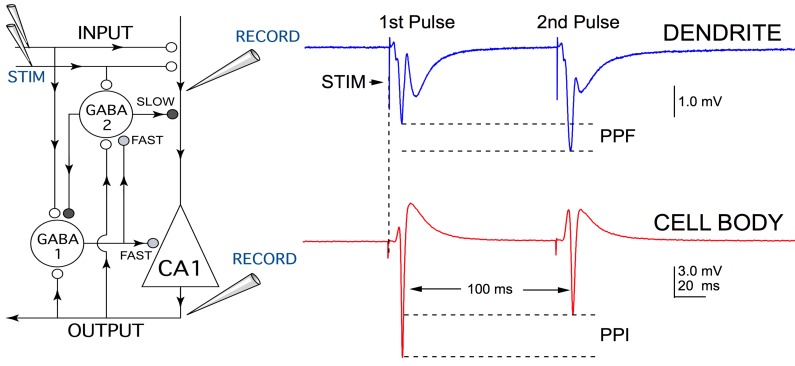
Inhibitory circuitry of the CA1 region. The panel on the left shows a schematic of the neuronal circuitry involved in the CA1 region of the hippocampus. The open circles represent glutaminergic, excitatory synapses and the closed darkened circles depict GABAergic, inhibitory synapses. Stimulation is sent in pulses, 100 ms apart (STIM) and recordings from different positions in the circuit exhibit different output responses. Recording from the dendritic (top) level presents as paired pulse potentiation (PPF), as a result of enhanced glutaminergic transmitter release on the second stimulus pulse. For the electrophysiological experiments discussed in this paper, a recording electrode was placed in the cell body region (bottom), as represented by the triangular shape labeled as CA1. Here, GABAA inhibition comes into play resulting in paired pulse inhibition (PPI) of the second population spike. The PPI ratio is calculated by dividing the amplitude of the first population spike by the amplitude of the second population spike.

To evoke population spikes (PS), the brain slices were stimulated at the Schaffer-collateral fibers (Figure 1) using a bipolar tungsten microelectrode (Fredreick Haer, Brunswick, ME). The population spike responses from CA1 neurons were recorded by placing a micropipette recording electrode filled with ACSF at the stratum oriens and stratum pyramidale border (Figure 2). Paired pulse recordings of population spikes were conducted at 10 ms (fast) and 100 ms (slow) interpulse intervals corresponding to two different types of GABA receptor mediated inhibitory activities, GABAA slow and GABAA fast (Figure 2).

 

At least eight slices were harvested from each animal and population spike recordings were averaged. The experimentors as well as the surgeon who harvested the brain slices were blinded for the experiments and only during analysis period was it known which animals corresponded to sham vs irradiated subjects. Data were collected, analyzed, and stored using software running under the Igor Pro data analysis package (Mac OS 10/UNIX, Wavemetrics, OR).

### Immunohistochemistry

Irradiated or sham-operated rats were deeply anesthetized and perfused transcranially with cold 0.9% saline, followed by four percent paraformaldehyde in phosphate-buffered saline (PBS) (pH 7.4) 24 or 48 hours after 60 Gy gamma radiation, n = 6 in each group. The brains were kept in four percent paraformaldehyde in PBS for three days and then cut into 50 μm coronal sections with a vibratome (VT1000S, Leica Microsystems, Wetzlar, Germany). Free-floating sections were washed in PBS and then treated with one percent H_2_O_2_ for 20 min. Nonspecific binding was prevented by incubating the sections for one hour in five percent normal goat serum in PBS containing 0.3% Triton X-100. The sections were incubated overnight at 4°C with various primary antibodies. After being washed three times in PBS, the sections were then incubated for one hour at room temperature in a relevant secondary antibody solution made up in PBS. The sections were washed and mounted on slides with Vectashield mounting medium for fluorescence containing 4′,6-diamidino-2-phenylindole (DAPI) (Vector Laboratories, Inc. Burlingame, CA) and studied by fluorescence microscopy.

### Data and statistical analysis

Average first population spike amplitudes (from the 10 minute baselines) were calculated for sham and irradiated slice recordings. Paired-pulse inhibition (PPI) was examined by calculating the PPI ratio. The PPI ratio is calculated by dividing the average amplitude of the first population spike by the average amplitude of the second population spike. A post analysis of variance (post-ANOVA) Tukey test (Igor Pro; Wavemetrics) was used to examine the statistical significance for each comparison made during the analysis period.

## Results

### Effects on synaptic transmission

**Figure 3:**
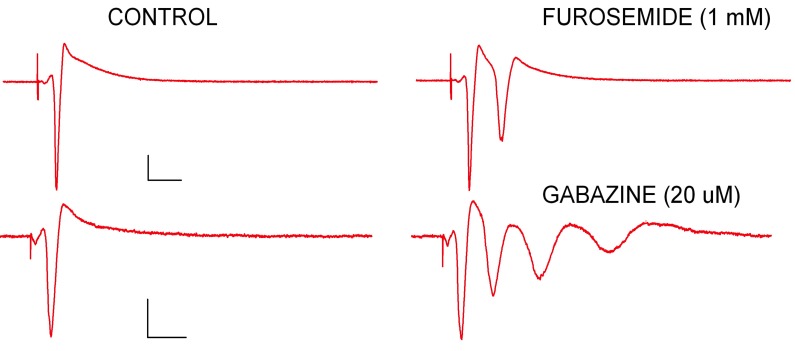
Two forms of GABA inhibition can be pharmacologically isolated using selective antagonists for different types of GABA receptors. Furosemide, a subunit-specific antagonist that exclusively blocks GABAA fast IPSCs changes the normal single population spike (PS) discharge into a pair of spikes, showing that GABAA fast inhibition comes on quickly (< 2 ms) but lasts only a short time (< 10 ms) and is responsible for limiting CA1 discharge to a single spike. Gabazine, an antagonist for GABAA fast and slow synaptic inhibition, results in multiple PSs lasting several hundred ms, showing that slow inhibition starts at ~ 5 ms and limits CA1 discharge over the time course studied in the present experiments (100 ms).

CA1 pyramidal neurons are controlled by several forms of GABA-mediated inhibiton that limit discharge activity to only a few milliseconds (GABAA fast) or for several hundred milliseconds (GABAA slow), or control the overall excitability of CA1 neurons (GABAA tonic receptors) [25-26]. Recording paired pulse inhibition of population spike (PS) responses provides a measure for each of these. Radiation effects on tonic inhibition would be seen as a change in PS amplitude. If tonic inhibition was reduced, then amplitudes would increase. Paired pulse inhibition of the second PS at 100 ms intervals provides a measure of GABAA slow inhibition (Figure 2). If GABAA fast inhibition was reduced, a secondary spike would appear (Figure 3).

Furosomide, a subtype-specific GABAA antagonist, has been shown to block GABAA fast induced pluripotent stem cells (IPSCs) [25-26], and it does so by acting on postsynaptic receptors. With the application of the GABAA fast blocker furosemide (1 mM), an extra, secondary PS is observed within 10 ms after the first PS (Figure 3). Blocking GABAA slow inhibition with gabazine (20 µM), in contrast, creates multiple PSs lasting over 100 ms (Figure 3). Gabazine has been previously characterized as being a competitive antagonist of GABAA receptors. Furthermore, Gabazine has a unique pharmacological property of blocking only phasic inhibitory postsynaptic currents (including GABAA fast and GABAA slow) but does not impact GABAergic tonic currents in CA1 neurons [27].

**Figure 4:**
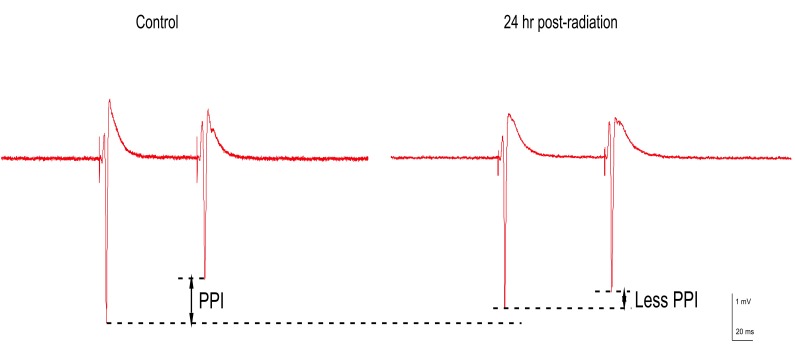
Synaptic responses comparing sham and irradiated recordings from brain slices. Population spike recording obtained from a sham animal (left) and a recording from a slice harvested from a rat 24 hours after 60 Gy gamma radiation (right). Gamma radiation depressed GABAA slow inhibition as evidenced by the reduction in the PPI level. There was also a small decrease in the first pulse responses, with no evidence of a significant reduction of GABAA fast inhibition (no secondary PS response).

**Figure 5:**
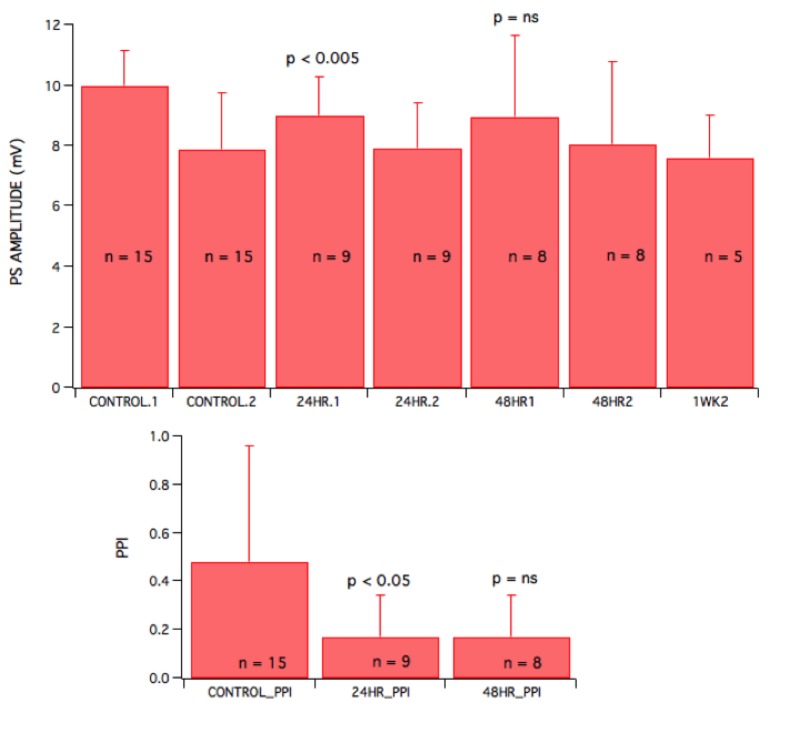
Gamma radiation of 60 Gy produced a small but statistically significant depression of GABAA slow-mediated inhibition 24 hours after irradiation that recovered after 48 hours and one week. Amplitude of population spikes in mV for first PS (for CONTROL.1, 24 hr, 48 hr and one week slices) as well as second PS (for CONTROL.2 and 24 hr slices). Combined data shows a decrease in the first spike amplitude for 24 hr, 48 hr, and one week animals after irradiation. However, when compared to sham animal data, only 24 hr rats had a significant decrease (p = 0.002; ANOVA), while the decrease of first PS seen in 48 hr and one week animals were not significant. There was no further significant decrease of the first PS seen for 48 hr and one week animals when compared to animals observed 24 hr after radiation. Average ratios of first and second spikes to assess amount of inhibition via PPI values were determined for control, 24 hr and 48 hr rats (Bottom). A small but significant decrease (14.4% change; p = 0.035 ; n = 9) of PPI was noted in 24 hr animals when compared to control. The apparent continued decrease of PPI in 48 hr rats was not significant (p = 0.11). Error bars represent the standard deviation of the mean.

Raw signals from field recordings of slices harvested from sham and irradiated animals were compared, and effects on GABAA synaptic inhibition were tested. GABAA mediated inhibitory transmission was depressed 24 hours after irradiation (Figure 4). However, unlike when gabazine or furosemide were added, secondary, multiple or prolonged PS recordings were not present in the 24 hr group. Depressed GABAA slow inhibition was also observed in slices harvested 24 hours after gamma irradiation, evident as a 14.4% decrease in PPI when compared to sham animals (p = 0.035, n = 9) (Figure 5). Although, 48 hr animals also exhibited a decrease in PPI, it did not achieve insignificances (p > 0.05) in the sample group we compared. There was a small, but significant decrease (p = 0.002, n = 9) of the first population spike in 24 hr recordings when compared to sham irradiated brains. Again, recovery of the first PS was observed in both 48 hr (insignificant decrease with p > 0.05) and one week groups.

 

### Effects on anatomy and immunohistochemistry

**Figure 6:**
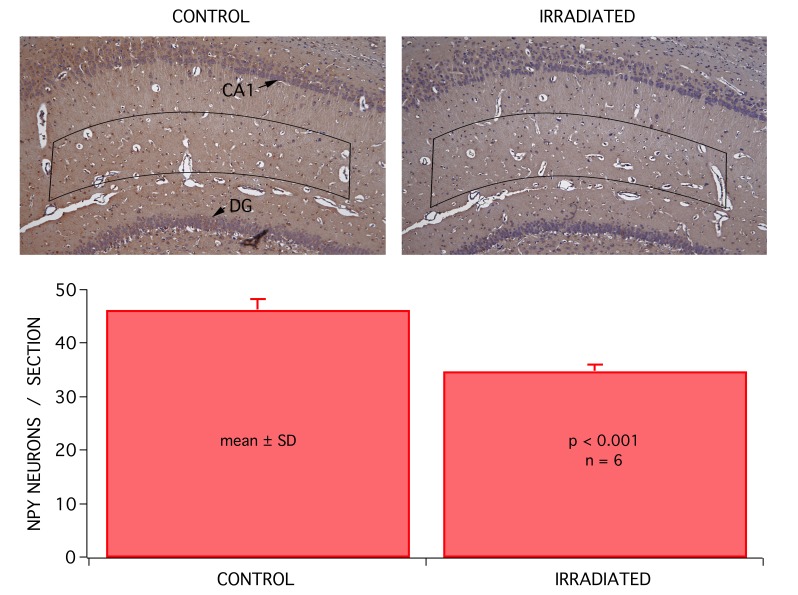
There was little or no apparent change in the overall anatomy of brain tissue in the CA1 region of the hippocampus (shown), or in any other brain region at the gross anatomy level. Detailed histological analysis also showed little or no change in morphology, but immunohistochemistry revealed a small decrease in neuropeptide Y (NPY) expressing GABAergic interneurons. The number of NPY neurons present per section of the hippocampus in the CA1 region of sham animals (n = 6) and rats irradiated 24 hr (n = 6) prior to brain slice harvesting were analyzed. There was a significant decrease (p < 0.001) in the number of NPY positive cells in irradiated rats when compared to sham (CONTROL) animals.

The overall appearance of brain slices was the same in control and irradiated animals. Similarly, there was no obvious difference at the microscopic level in irradiated animal tissue (Figure 6). Immunohistochemical analysis of hippocampal slices were conducted on brain slices prepared from sham and irradiated groups. Levels of distinct proteins were compared and neuropeptide Y (NPY) positive neurons exhibited a significant reduction. Compared to the sham group, the number of NPY expressed in neurons present per section of hippocampus in the CA1 region was significantly lower in 24 hr groups (p < 0.001; ANOVA, n = 6; Figure 6).

## Discussion

Although many studies have looked at the behavioral and cognitive performance deficits following irradiation, only a few papers have explored the neuronal modifications at the synaptic and individual neuronal level. Even fewer have tried to examine how the anatomical changes correspond to the neuronal responses by combining immunochemical and electrophysiological investigations.

The electrophysiological results presented here do not demonstrate any major changes for PS responses at any post-irradiation time point. Exposure to 60Gy of irradiation had very little effect on synaptic transmission, neuronal excitability, or synaptic inhibition. Radiation also did not appear to alter GABAA tonic or GABAA fast inhibition, but produced a statistically significant reduction in synaptic inhibition seen at 100 ms, indicating a selective depression of GABAA slow inhibition. This was also evident in the reduction in the PPI values seen in irradiated rats. Overall, these were relatively minor effects and are consistent with earlier studies suggesting that gamma radiation has only minor and transient effects on brain tissue at clinically relevant doses (< 60 Gy). For example, a study looking at hippocampal neuron numbers showed that they remained unchanged one year after a rat was exposed to fractionated whole brain irradiation at a dose of 45 Gy applied twice a week for 4.5 weeks [7]. Although the magnitude of effects was relatively low, it could still have a physiological effect and can potentially be clinically meaningful. This idea of neuromodulation by changing neuronal behavior without affecting the physiological integrity of neuronal tissue has been of great interest to groups trying to use focally directed SRS to treat patients with various clinical disorders [28]. This review paper discussing a collection of studies looking at localized irradiation bringing treatment to patients suffering from such ailments as trigeminal neuralgia and epilepsy proposes that these minimal and mainly safe changes observed in radiomodulation can play a therapeutic role for other functional and behavioral disorders.

The results presented in this paper showed that following gamma radiation treatments, there were no physically-evident pathological effects; however, a subtle functional depression in circuit level inhibition was evident. The reduced expression of NPY in interneurons could help account for this functional depression, since NPY interneurons are known to be GABAergic and to mediate GABAA slow inhibition [29]. It is ulikely that irradiation harmed NPY interneurons, since the effect occurred too soon for this, but it is more likely that a reduction in NPY expression occurred. This change, although temporary (24 hr group), could contribute to antidepressant effects of radiation. In fact it may give us a glimpse into how radiation works in a clinical setting for use of a treatment for patients with functional conditions including major depressive disorder. Similar to what was observed in our study, antidepressants produce a statistically significant depression of GABAA slow-mediated inhibition in the rat hippocampus [30].

## Conclusions

Radiomodulation of neuronal circuitry was evident as a small but statiscally significant decrease in GABA-mediated slow synaptic inhibition.
